# Personality assimilation across species: enfacing an ape reduces own intelligence and increases emotion attribution to apes

**DOI:** 10.1007/s00426-018-1048-x

**Published:** 2018-07-02

**Authors:** Ke Ma, Roberta Sellaro, Bernhard Hommel

**Affiliations:** 1 Cognitive Psychology Unit, Institute for Psychological Research and Leiden Institute for Brain and Cognition, Leiden University, Wassenaarseweg 52, 2333 AK Leiden, The Netherlands; 2 Present Address: Key Laboratory of Personality and Cognition, Faculty of Psychological Science, Southwest University, Beibei, Chongqing, China

**Keywords:** Self-representation, Illusory conjunction, Sense of ownership, Attribution, Intelligence

## Abstract

Seeing another person’s face while that face and one’s own face are stroked synchronously or controlling a virtual face by moving one’s own induces the illusion that the other face has become a part of oneself—the enfacement effect. Here, we demonstrate that humans can enface even members of another species and that this enfacement promotes “feature migration” in terms of intelligence and emotional attribution from the representation of other to the representation of oneself, and vice versa. We presented participants with a virtual human face moving in or out of sync with their own face, and then morphed it into an ape face. Participants tended to perceive the ape face as their own in the sync condition, as indicated by body-ownership and inclusion-of-others-in-the-self ratings. More interestingly, synchrony also reduced performance in a fluid-intelligence task and increased the willingness to attribute emotions to apes. These observations, which fully replicated in another experiment, fit with the idea that self and other are represented in terms of feature codes, just like non-social events (as implied by the Theory of Event Coding), so that representational self–other overlap invites illusory conjunctions of features from one representation to the other.

## Introduction

The ability to differentiate one’s own body from others’ is commonly thought to rely on continuous body representations (Gallagher 2000; Tsakiris, 2008; Lenggenhager et al., 2007), which, however, can be updated and adjusted to the present situation (e.g., Graziano & Botvinick, 2002). For example, the rubber hand illusion (RHI) shows that people perceive ownership for a rubber hand lying in front of them if the rubber hand and their real hand are stroked synchronously (Botvinick & Cohen, 1998). Similarly, the virtual hand illusion (VHI) demonstrates that people perceive ownership for a virtual hand if they can operate its movements by moving their own hand (Slater et al., 2008). Even more important for our present purposes, people tend to perceive the face of another human as their own if it is stroked in synchrony with their own face—the enfacement illusion (Tsakiris, 2008; Porciello, Bufalari, Minio-Paluello, Di Pace & Aglioti, in press).

Research suggests that embodying the body or face of another person does not only tend to diminish self–other discrimination, but also to invite what Ma, Sellaro, Lippelt, and Hommel (2016) called “feature migration”. This hypothetical process is derived from recent attempts to apply the Theory of Event Coding (TEC: Hommel, Müsseler, Aschersleben & Prinz, 2001) to the representation of self and others (Hommel, Colzato & van den Wildenberg, 2009). The idea is that people represent themselves and others just like other perceptual events, namely, in terms of integrated networks of sensorimotor feature codes (event files: Hommel, 2004) representing all discriminable features an event or person consists of, such as physical attributes, affective responses, control states, and covert and overt actions associated with a given event. Importantly, given that feature codes are integrated and bound together, they tend to be retrieved as a whole when one of the features of a given event is encountered. Moreover, the activation of feature codes is regulated by an “intentional weighting” process that gives more weight to attended or task-relevant features (Memelink & Hommel, 2013). Accordingly, focusing on common or discriminating features can reduce or increase the degree to which self and other are perceived as different, respectively (Colzato, de Bruijn, & Hommel, 2012). Importantly for present purposes, to the degree that the representation of self and other is perceived as part of the same event, features of one’s representation can “migrate” to the representation of the other (Treisman & Gelade, 1980)—i.e., features of one event can become part of an “illusory conjunction” with the other.

Indeed, Ma et al. (2016) observed that controlling the movements of a virtual face by moving one’s own lead to the migration of mood: participants were significantly happier and performed better in a mood-sensitive brainstorming task when controlling a smiling compared to a neutral face, but only if virtual and actual face moved in synchrony. In terms of TEC, experiencing a virtual happy face as being part of oneself caused participants to confuse their own features and states with the features and states of the virtual face to the extent that affective features of the virtual face (i.e., the smile) became assimilated with participants’ self-representation. Other studies suggest that embodying another person’s hand, face, or body affects people’s attitudes towards the other or the other’s social group (for a review, see Maister, Slater, Sanchez-Vives & Tsakiris, 2015). For example, occupying a virtual child body facilitates combining the self with child-like attributes in an implicit-association test (Banakou, Groten, & Slater, 2013), owning a dark-skinned rubber hand or a black avatar reduces implicit racial bias of light-skinned for dark-skinned people (Maister, Sebanz, Knoblich, & Tsakiris, 2013; Farmer, Maister, & Tsakiris, 2013; Peck, Seinfeld, Aglioti, & Slater, 2013; but see Estudillo & Bindemann, 2016), embodying avatars of old people, compared to young people, reduces negative stereotyping of the elderly (Yee & Bailenson, 2006), and placing participants in avatars with a superhero ability promotes helping behavior (Rosenberg, Baughman, & Bailenson, 2013). These observations suggest that feature migration can change both attitudes and behavior, which fits with the idea that feature codes are sensorimotor in nature, in the sense that they are activated by sensory information (i.e., perception) and can regulate both perception and overt/covert behavior (Hommel et al., 2001).

So far, the impact of the embodiment of others was almost exclusively restricted to human bodies or body parts. Assuming a permanent body representation, this makes sense: candidate effectors should be accepted as possible body parts only to the degree that they are similar to parts that this representation entails (Tsakiris, Carpenter, James, & Fotopoulou, 2010). However, there is growing evidence that perceived body ownership may be even more flexible than hitherto assumed. For instance, recent studies have shown that people can experience ownership over avatars that are shaped differently from them, including not only avatars of different gender (Slater et al., 2010), race (Maister, Sebanz, Knoblich, & Tsakiris, 2013; Farmer, Maister, & Tsakiris, 2013; Peck, Seinfeld, Aglioti, & Slater, 2013; Bufalari et al., 2014) and age (Oh et al., 2016; Banakou, Groten, & Slater, 2013; Hershfield et al., 2011; Yee & Bailenson, 2006), but also avatars with very long arms (Kilteni et al., 2012), with abnormally large and small bodies (van der Hoort, Guterstam, & Ehrsson, 2011; see also Piryankova et al., 2014), with tails (Steptoe, Steed & Slater, 2013), and with amputated body parts (Kilteni et al., 2016). Going a step further, we recently demonstrated ownership for visual objects without any obvious similarity to body parts, such as balloons and rectangles (Ma & Hommel, 2015a).

Taken together, the aforementioned findings make it plausible to expect illusions of this sort to be also demonstrated across species. To the best of our knowledge, only one recent study has addressed this issue (Ahn et al., 2016). Ahn et al. used immersive virtual reality to make participants viscerally experience the life through the eyes of a cow or coral in an acidifying reef. When embodying a cow, participants walked in a virtual pasture on all fours, while eating feed and drinking water, they were goaded with a virtual cattle prod and finally they were loaded onto a truck; when embodying a coral on a rocky reef, participants were let to experience coral suffering from ocean acidification by seeing, hearing and feeling the reef around them as well as their own body corrode. Results showed that participants embodying the cow or the piece of coral, compared to those who simply watched a video of these experiences, felt more connected with the nature and had more concerns about the environment.

In the present study, we were interested to see whether embodiment across species can be elicited also by simply letting participants control the movement of a virtual face of a member of another species. More importantly, we aimed to assess whether enfacing members of another species would induce self–other assimilation as observed for the embodiment of human body parts. To test that possibility, we designed an ape face, which is clearly discriminable from a human face but still keeps some degree of resemblance. To induce identification with this face we used a dynamic enfacement paradigm (see Ma et al., 2016), in which participants could move the virtual face either synchronously or asynchronously.

Sforza et al. (2010) reported findings showing that questionnaire ratings for enfacement illusions tend to be low on average, compared to ratings obtained with the RHI and embodiment illusions. This suggests that the subjective experience of enfacement can be harder to obtain for faces than for other body parts, probably because facial identity is at the core of the sense of the self (Tsakiris, 2008). Being confronted with the face of a member of another species is not unlikely to cause feelings of strangeness and irritation, similar to the ones reported during virtual body illusions (Lenggenhager et al., 2007), which might work against enfacement. Therefore, to reduce the risk of incurring in such irritations and to maximize the virtual enfacement illusion, we used a morphing procedure in which a synchronously- or asynchronously-moving virtual human face slowly morphed into an ape face. Note that we applied this technique to both synchrony conditions, so that possible effects of synchrony would be corrected for possible effects on the morphing procedure itself.

Building on earlier findings, we expected a synchronously-moving virtual face to increase perceived ownership, as measured by a standard enfacement questionnaire, and increase self–ape similarity. Crucially, we hypothesized that ownership, in turn, should facilitate feature migration between the representations of self and ape. Importantly, according to TEC logic, feature migration of this sort can involve all kind of features and can occur from the representation of other to the representation of oneself, and vice versa.

We tested two features that we considered particularly diagnostic. First, we had participants to undergo the Standard Progressive Matrices (SPM) test (Raven, 1938), which assesses fluid intelligence. Previous studies have shown that people may conform to the expected attitudes and behavior of the avatar they are represented by, without necessarily being aware of that (i.e., the Proteus effect; e.g., Yee & Bailenson, 2007; Yee, Bailenson, & Ducheneaut, 2009; Peña, Hancock, & Merola, 2009). For instance, participants have been found to behave more confidentially and friendlier with strangers or to behave more aggressively during a negotiation when controlling attractive and tall avatars, respectively (Yee & Bailenson, 2007; see also Yee, Bailenson, & Ducheneaut, 2009). Similarly, it has been found that participants represented by avatars dressed in Ku Klux Klan outfits reported more negative and aggressive thoughts than those dressed as doctors (Peña et al., 2009). Taken together, these findings are consistent with previous literature showing that trait concepts and stereotypes become active automatically in the presence of relevant behavior or stereotyped-group features (i.e., through perception), and can cause people to behave consistently with the activated stereotypes (Chartrand & Bargh, 1999). Therefore, considering the common stereotype that humans are more intelligent than non-human animals, we predicted that enfacing and, thus, in some sense becoming an ape should reduce intelligence and result in a lower score in the SPM test. Note that we relied on, but did not directly test this stereotype in our participants because the assessment would have primed the respective stereotype (cf., Memelink & Hommel, 2013), thus introducing a confound and rendering our test less diagnostic.

Second, we tested participants by means of an emotion rating task assessing to which degree participants would attribute particular emotions to humans and apes (Demoulin et al., 2004). As not all emotions are assumed to be shared among humans and other animals, we expected that more emotions would be attributed to humans than to apes, but that this difference would be reduced or eliminated after enfacing an ape. Indeed, there is evidence that embodiment illusions can affect people’s perception of and beliefs towards the embodied other, and that positive self-like associations can be extended to the embodied others (Maister et al., 2015). Therefore, given that people are biased to maintain a positive self-image (Tajfel & Turner, 1986), increased self–ape similarity should cause participants to attribute a higher ability to experience emotions to apes. In contrast, attribution of emotions to humans is not expected to be affected by the enfacement manipulation. Note that possible synchronicity-induced changes in the attribution of emotions to apes may be restricted to emotions that are considered to be not uniquely human (i.e., primary emotions; Epstein, 1984), or may concern primary and secondary emotions alike. Whereas the former case would indicate that participants have judged apes from their human perspective, the latter case would indicate that they have judged apes from the perspective of an ape feeling like a human.

## Methods

### Participants

Given the unpredictable effect sizes, the sample was chosen to double our lab standard for novel manipulations (20/group; see Simmons, Nelson & Simonsohn, 2011). 52 volunteers participated in the study for course credit or pay but 12 of them were excluded due to technical problems—thus leaving 40 participants (mean age 20.88 years, SD = 2.83 years, range 18–29 years; seven males). We used the department’s standard advertisement system and accepted all participants registering in the first (and only) wave. Written informed consent was obtained from all participants before the experiment. Participants were naive as to the purposes of the experiment. The study conformed to the ethical standards of the declaration of Helsinki and the protocol was approved by the local research ethics committee.

### Experimental setup

**Fig. 1:**
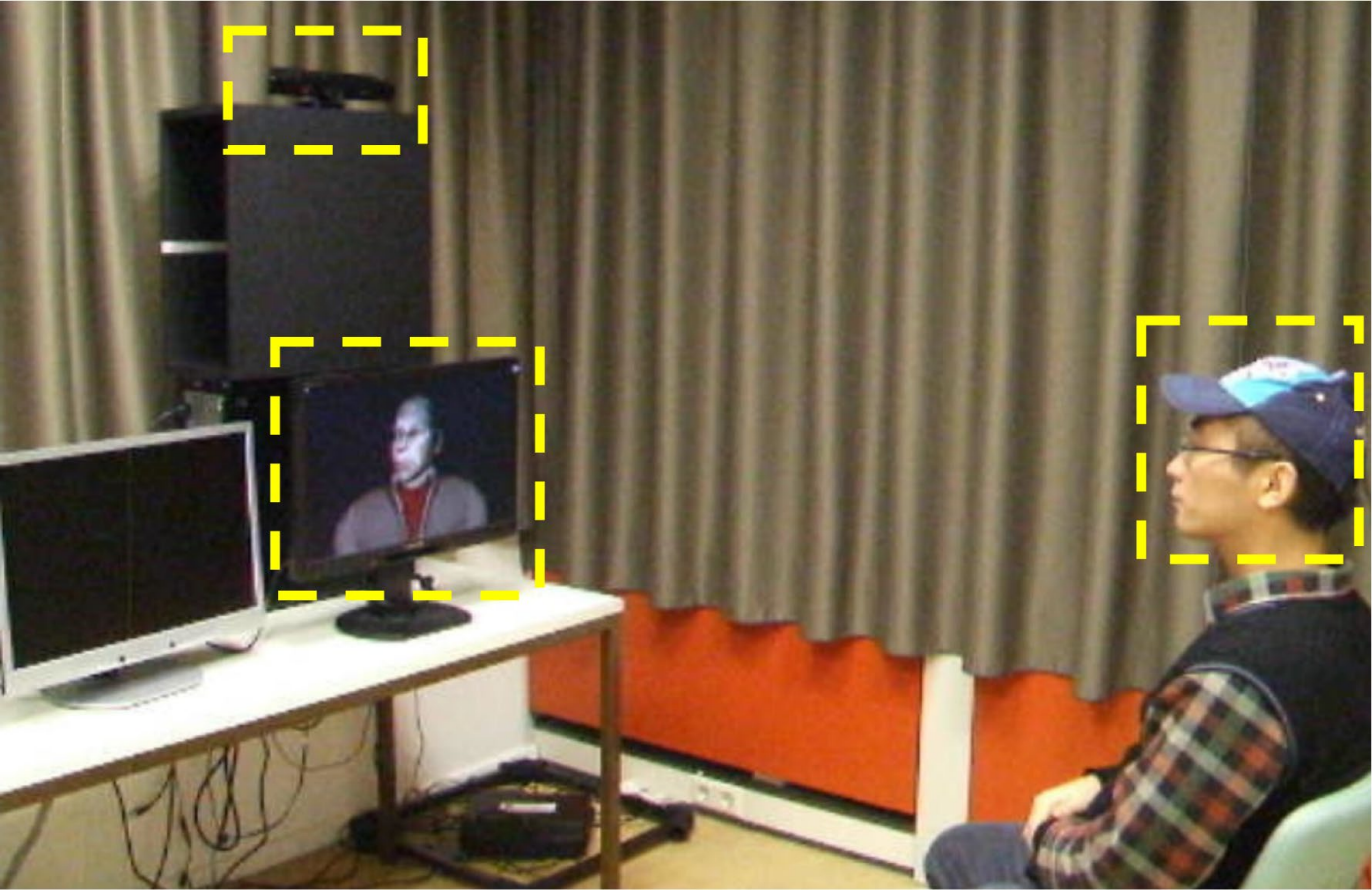
The experimental setup. The participant’s facial movements were monitored by means of a Kinect system (recording frame rate = 30 Hz) and an Intersense orientation tracker (update rate = 180 Hz). The Kinect system (see upper left yellow frame) was located behind and above the computer screen showing the virtual face (see lower left yellow frame). Participants sat at about 2 m from the Kinect system, which requires a minimum distance of 1.8 m to recognize human movements. Participants (here the first author) wore a cap with an orientation tracker attached on it (see right yellow frame). Computer tasks (i.e., the emotion rating and the Raven’s Standard Progressive Matrices tasks) were presented on a second screen located next to the screen showing the virtual face

**Fig. 2:**
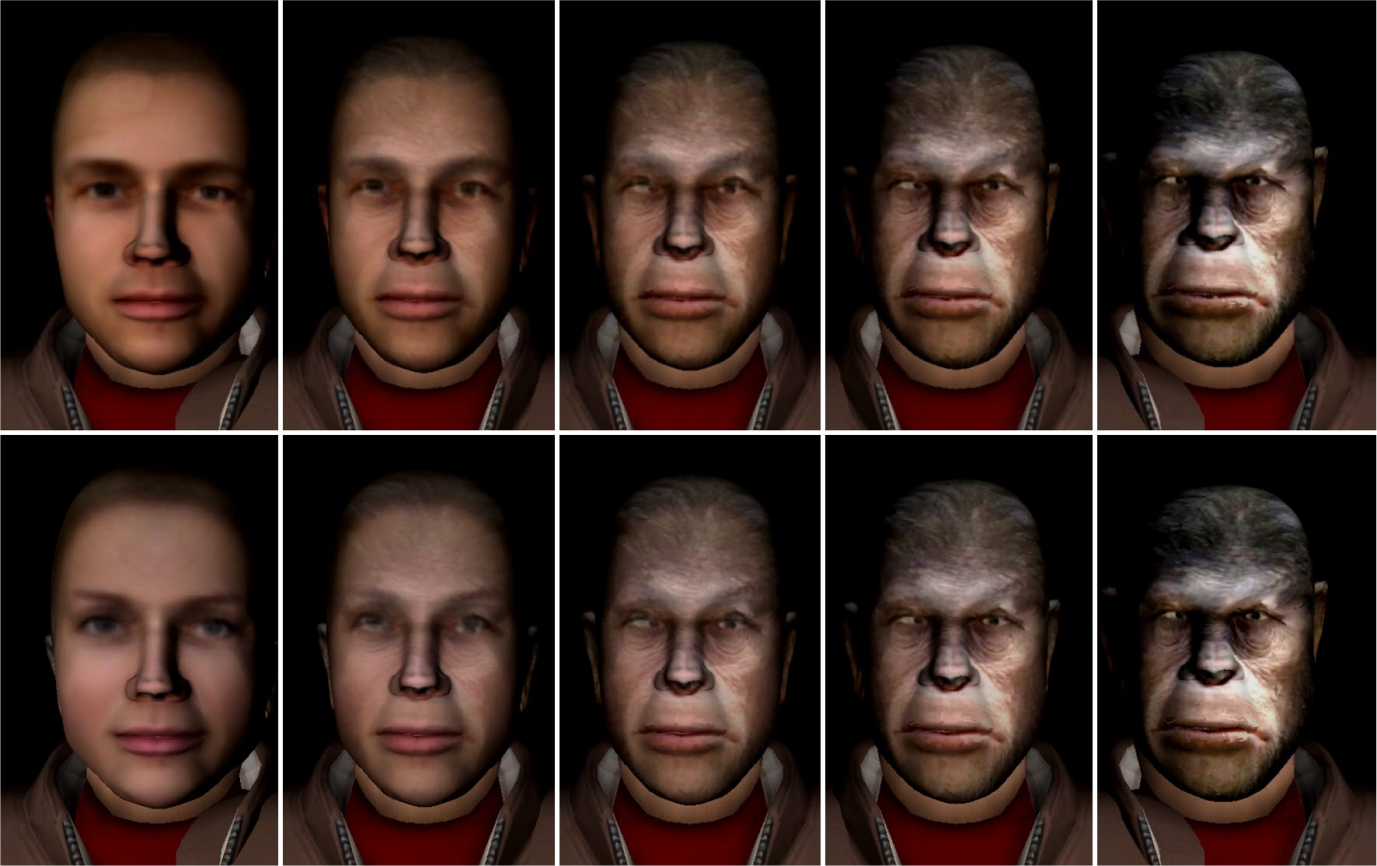
Some representative images resulting from the morphing procedure applied to male (upper panel) and female (lower panel) faces. From left to right, 100% human and 0% ape, 75% human and 25% ape, 50% human and 50% ape, 25% human and 75% ape, and 0% human and 100% ape. The resulting 100% ape face was identical regardless of whether the male or the female face was morphed

Figure 1 shows the basic setup. The virtual faces were constructed and controlled by means of virtual reality environment software (Vizard and FAAST; Suma et al., 2013). We used Vizard to build three three-dimensional virtual faces, one for each gender, based on average Caucasian faces (e.g., Jones et al., 2006) and one for the ape face. A computerized morphing procedure was used to gradually merge the human and the ape faces (see Fig. 2). By integrating Kinect, Intersense, FAAST, and Vizard, our setup allowed participants to freely move or rotate their own face to control the movement or rotation of the virtual face, with a latency of about 40 ms—a value far below the 300-ms threshold proposed by Shimada, Fukuda, and Hiraki (2009) as the critical time window allowing for the occurrence of multi-sensory integration processes constituting self-body representation.

### Measures

#### Enfacement questionnaire

Four items were adopted from the standard RHI questionnaire (Botvinick & Cohen, 1998) and enfacement studies (Tajadura-Jiménez, Grehl, S., & Tsakiris, 2012; Sforza et al., 2010). Q1 (“I felt the ape face was my own face”) directly addresses perceived ownership, Q2 (“It seemed like I was sensing the movement of my face in the location where the ape face on the screen was”) is a location-related ownership item that is sometimes aggregated with direct ownership to assess perceived ownership properly (Kalckert & Ehrsson, 2014), Q3 (“It seemed like my own face began to resemble the ape face on the screen”) assesses perceived appearance similarity, a possible correlate of ownership (Tajadura-Jiménez et al., 2012), and Q4 (“I could control the ape face”) perceived agency, which according to Ma and Hommel (2015b) predict ownership in participant-active setups. For each item, participants chose a score on a Likert scale ranging from 1 (“strongly disagree”) to 7 (“strongly agree”). Note that we did not use the “control” questions of the RHI questionnaire, as in our previous studies (Ma & Hommel, 2015a, 2015b) we found evidence suggesting that, for illusions created using VR, synchrony is likely to affect responses to control questions as well. This is likely because dynamic manipulations, compared to the static ones, can make all self-perception aspects perceivable or salient, thereby producing measurable synchrony effects on all items relating to any aspect of self-perception that can correlate with ownership and agency (cf., Ma & Hommel, 2015b).

#### Including others in the self (IOS) scale

A variant of the IOS scale (Aron, Aron, & Smollan, 1992) assessed subjective aspects of self–other integration. The scale consists of seven Venn diagram-like pairs of circles representing varying degrees of self–other overlap (i.e., subjective self–other integration). Participants are to choose the overlap that they think represents best the degree to which the virtual face looks like their own, how familiar it feels to them.

#### Raven’s standard progressive matrices (SPM)

SPMs (Raven, 1938) assessed participants’ fluid intelligence. The test consists of 60 trials of increasing difficulty. Participants either received the even 30 trials on the first experimental condition and the odd 30 trials on the second experimental condition or vice versa (counterbalanced across participants).

#### Emotion rating task

The emotion rating task was adopted from De Dreu et al. (2011). Participants were asked to rate, on a Likert scale ranging from 1 (not at all) to 7 (very much), how much humans and apes are able to experience 12 emotions (Epstein, 1984; Demoulin et al., 2004), 6 primary (affection, pleasure, attraction, fear, exhaustion, and pain) and 6 secondary emotions (admiration, hope, surprise, embarrassment, contempt, humiliation). Each participant performed two task blocks, one in which emotions were rated for Human and one in which they were rated for Ape. The order of the two blocks was counterbalanced across participants, and within each block emotions were presented in a random order.

### Procedure

After having read and signed the informed consent, participants were seated in front of a computer monitor and to wear the cap (as shown in Fig. 1). Each participant underwent two synchrony (synchronous and the asynchronous) conditions in counterbalanced order. During both synchrony conditions, the participants actively operated the virtual face for 3 min by freely displacing or rotating their own face, which led to corresponding displacement or rotation movements of the virtual face. The 3-min interval was divided into three parts. During the first 30 s, participants were presented with either the male or the female virtual face (depending on their gender), which was 100% human. Then, for the next 120 s, the human face was morphed into the ape face (from 100% human–0% ape to 0% human–100% ape, in steps of 0.83% every second, for a total of 120 morphs, plus the two corresponding mapped texture pictures, each for male or female participants; see Fig. 2 for representative images resulting from the morphing procedure). For the remaining 30 s, the viewed face was 100% ape.

The only difference between the two conditions pertained to the temporal delay between people’s own movements and that of the virtual face, which—excluding the 40-ms time delay caused by the equipment—was either 0 s (= synchronous) or 3 s (= asynchronous). Immediately after each synchrony condition, participants answered the enfacement questionnaire, responded to the IOS scale, performed the emotion rating and eventually the Raven task. We preferred not to counterbalance the order of the Raven and the emotion rating tasks for the following reason. Research has shown that performing a demanding cognitive task is likely to reduce self-control (i.e., it can cause ego-depletion; Baumeister, Bratslavsky, Muraven, & Tice, 1998), and evidence exists that lack of self-control can affect interpersonal perception (e.g., Vohs & Ciarocco, 2004; Govorun, & Payne, 2006). Therefore, to avoid possible confounding effects resulting from ego-depletion we preferred to have participants perform the most demanding task (i.e., the Raven task) after the emotion ratings task. All participants were asked to take a 5-min break between the two synchrony conditions.

## Results

### Enfacement questionnaire

Shapiro–Wilk test revealed that the distribution of the differences deviated significantly from the normal distribution for Q2, Q3 and Q4 (*p*_s_ ≤ 0.04), but not for Q1 (*p* = 0.06). Because the data were not normally distributed for three out of four questionnaire items, the non-parametric Wilcoxon signed-rank test was used to compare ratings across the two synchrony conditions. For non-symmetrically shaped distributions values resulting from the non-parametric sign test are reported. Analyses showed that synchrony was significant for all items (Q1: *Z* = − 3.85, *p* < 0.001, *r* = 0.61; Q2: *Z* = − 4.50, *p* < 0.001, *r* = 0.71; Q3: *Z* = − 3.47, *p* < 0.001, *r* = 0.55; Q4: *Z* = − 4.53, *p* < 0.001, *r* = 0.72), with participants experiencing more ownership, similarity, and agency in the synchronous than in the asynchronous condition. Hence, we successfully induced a virtual enfacement illusion for the ape face. Table 1 provides participants’ ratings (i.e., median and range in parentheses) separately for each item and for the aggregated ownership items (Q1–Q2) as a function of synchrony condition.

**Table 1:** Overview of participants’ performance for all dependent measures as a function of synchrony

Measure	Experiment		Replication	
	Synchrony	Asynchrony	Synchrony	Asynchrony
Enfacement questionnaire (median and range)				
Direct ownership (Q1)^a^	4.0 (6)	2.0 (6)	3.0 (5)	2.0 (6)
Location (Q2)^a^	5.5 (6)	3.0 (6)	5.0 (6)	2.0 (5)
Aggregated ownership (Q1-Q2)	4.5 (6)	2.75 (6)	4.0 (5)	2.5 (5.5)
Similarity (Q3)^a^	3.0 (6)	2.0 (5)	2.0 (6)	2.0 (5)
Agency (Q4)^a^	6.0 (6)	4.0 (6)	6.5 (6)	4.5 (6)
IOS^a^ (median and range)	4.5 (6)	3.0 (6)	4.0 (5)	3.0 (5)
Raven^a^ (median and range)	25.0 (13)	26.0 (12)	25.0 (9)	26.0 (10)
Emotion rating (mean and standard error of the mean)				
Human (primary)^ns^	6.63 (0.07)	6.65 (0.06)	6.71 (0.07)	6.69 (0.08)
Human (secondary)^ns^	6.58 (0.08)	6.63 (0.09)	6.48 (0.12)	6.55 (0.11)
Ape (primary)^a^	6.07 (0.10)	5.95 (0.11)	5.71 (0.14)	5.36 (0.22)
Ape (secondary)^a^	4.78 (0.19)	4.50 (0.22)	4.33 (0.19)	3.89 (0.24)

Median values and range (in parentheses) are reported for the enfacement questionnaire items, IOS and Raven, whereas means and standard error of the mean (in parentheses) are reported for the emotion rating task. Significant and non-significant synchrony effects are indicated by “^a^” and “ns”, respectively. Columns 2 and 3 show results for the original experiment, and columns 4 and 5 for the replication

### IOS

Shapiro–Wilk test revealed that the distribution of the differences deviated significantly from the normal distribution (*p* = 0.002). A Wilcoxon signed-rank test showed that the synchrony effect was significant, indicating that participants experienced greater overlap with the virtual face after the synchronous than after the asynchronous condition, *Z* = − 4.45, *p* < 0.001, *r* = 0.70, see Table 1.

### SPM

Shapiro–Wilk test revealed that the distribution of the differences deviated significantly from the normal distribution (*p* = 0.043). A sign test showed that the synchrony effect was significant, with a significant median decrease in Raven scores after the synchronous condition, compared to the asynchronous one, *Z* = − 2.03, *p* = 0.043, *r* = 0.32, see Table 1.

### Emotion ratings

Shapiro–Wilk test indicated that residuals were not normally distributed for the majority of factor level combinations (*p* < 0.05). However, due to the central limit theorem, the ANOVA can be considered robust to violations of normality (Glass, Peckham & Sanders, 1972; Harwell, Rubinstein, Hayes & Olds, 1992; Schmider, Ziegler, Danay, Beyer, & Bühner, 2010). Therefore, ratings were analyzed by means of a repeated-measure ANOVA with synchrony (synchronous vs. asynchronous), species (human vs. ape) and emotion type (primary vs. secondary) as within-participant factors. ANOVA revealed significant main effects of emotion type, *F*(1,39) = 65.39, *p* < 0.001, *ηp*2 = 0.63, and species, *F*(1,39) = 86.27, *p* < 0.001, *ηp*2 = 0.69: ratings were higher for primary than for secondary emotions (6.3 vs. 5.6) and, as expected, for humans than for apes (6.6 vs. 5.3). Moreover, a significant interaction involving the factor species and emotion type was found, *F*(1,39) = 71.07, *p* < 0.001, *ηp*2 = 0.65. Consistent with previous findings (Epstein, 1984; Demoulin et al., 2004), Bonferroni post hoc tests revealed that participants judged apes to be able to feel primary emotions to a greater extent than secondary emotions (6.0 vs. 4.6, *p* < 0.001), whereas no difference between primary and secondary emotions was found when rating human emotions (6.6 vs. 6.6, *p* = 1). More importantly, the interaction between synchrony and species was also significant, *F*(1,39) = 6.22, *p* = 0.017, *ηp*2 = 0.14. Bonferroni post hoc tests confirmed that participants attributed to apes a higher capacity to feel emotions in the synchronous than in the asynchronous condition (*p* = 0.03), whereas no difference between the two synchrony conditions was observed when rating human emotions (*p* = 1; see Table 1). No other significant sources of variance were observed, *F*_s_ ≤ 2.78, *p*_s_ ≥ 0.10.

## Replication

While the findings came out as expected, some of the effects were rather small numerically and just reached the significance level. To make sure that our conclusions are not based on spurious, non-reproducible findings, we conducted an exact replication study. 40 new participants were tested, and the statistical findings were exactly as in the first experiment: the synchrony effect was significant for all enfacement questionnaire items (Q1: *Z* = − 2.35, *p* = 0.02, *r* = 0.37; Q2: *Z* = − 5.13, *p* < 0.001, *r* = 0.81; Q3: *Z* = − 2.62, *p* = 0.01, *r* = 0.41; Q4: *Z* = − 3.85, *p* < 0.001, *r* = 0.61), IOS ratings, *Z* = − 3.40, *p* < 0.001, *r* = 0.54, Raven scores, *Z* = − 2.34, *p* = 0.02, *r* = 0.37, and a significant two-way interaction involving the factors synchrony and species was found for the emotion rating task, *F*(1,39) = 9.46, *p* = 0.004, *ηp*2 = 0.20, with synchronicity affecting emotion ratings when judging apes but not when judging humans (see Table 1).

## Correlations

Next, we combined the data of the two experiments to assess the relationship between perceived ownership and ownership-related characteristics (self–other similarity and agency), and between these factors and task (emotion ratings for Ape and Raven) performance. The degrees of ownership (as assessed by the aggregation of Q1–2), agency (Q4), and self–other similarity (IOS ratings) were computed by subtracting asynchronous ratings from synchronous ratings. Likewise, synchrony-induced changes in task performance were computed by subtracting emotion ratings for Ape and Raven scores observed after the asynchronous condition from those observed after the synchronous condition. We computed one-tailed Spearman correlations among changes in ownership, agency and IOS ratings, and synchrony-induced changes in task performance. Significant and positive correlations were observed between ownership and IOS changes, rho = 0.45, *p* < 0.001, ownership and agency changes, rho = 0.24, *p* = 0.017, and between agency and IOS changes, rho = 0.41, *p* < 0.001. Interestingly, emotion rating changes correlated positively with agency, rho = 0.40, *p* < 0.001, and IOS changes, rho = 0.29, *p* = 0.004, and negatively with Raven changes, *r* = − 0.26, *p* = 0.010. No other significant correlations were found, absolute rho values ≤ 0.09, *p*s ≥ 0.22.

## Discussion

This study set out to test whether embodiment can be demonstrated across species and whether this would induce self–other assimilation (and feature migration) in terms of intelligence and emotion attribution—as implied by our application of TEC (Hommel et al., 2001). The first question can be answered affirmatively: when the ape face moved in synchrony with the participants’ own face, they were more likely to perceive ownership for the former. It is true that ownership perception was not perfect, as the score fell into the midrange of the scale—a finding that is consistent with other studies of the virtual-hand (e.g., Ma & Hommel, 2015a, 2015b) and the virtual-face illusion (Ma et al., 2016; Tajadura-Jiménez et al., 2012; Sforza et al., 2010). This limitation notwithstanding, the fact that a short practice over 3 min was sufficient to affect one’s identification with another species—which most participants were likely to have little experience with—must be considered notable. Converging evidence supporting the conclusion that our participants identified with the virtual ape comes from the IOS ratings, which confirm that synchrony increased the perceived overlap between participant and ape. Hence, humans are able to identify with a member of another species, probably in a similar way as they can embody the hand or face of another human. This result is not unanticipated as it fits with previous claims that people’s self-construal is dynamic and sensitive to situational and cultural biases (Colzato et al., 2012; Kühnen & Oyserman, 2002), and that people are rather flexible regarding which objects and events they consider as being part of their body—as long as they can control the behavior of these objects and events (Ahn et al., 2016; Kilteni et al., 2016; Ma & Hommel, 2015a, b; Piryankova et al., 2014; Steptoe et al., 2013; Kilteni et al., 2012; van der Hoort et al., 2011). It is worth noting that, in the present study, to maximize the chance of eliciting a virtual enfacement illusion of an ape face, we made use of a morphing procedure in which a synchronously or asynchronously moving virtual human face slowly morphed into an ape face. As mentioned in the Introduction, such a choice was aimed at counteracting possible feelings of irritation and strangeness that could have arisen by confronting participants directly with the face of a member of another species. It remains to be established whether the morphing procedure was really necessary for the illusion to occur, or whether virtual enfacement of a member of a different species can also be obtained without such a procedure.

The second most important question can also be answered affirmatively: both the intelligence measure and the emotion attributions were affected by synchrony. As predicted from our TEC-based approach, perceiving oneself to own an ape face made people behave less intelligently; that is, participants tended to adopt the intellectual characteristics humans attribute to the species they perceived themselves to become a part of, thereby confirming previous findings documenting people’s tendency to adopt attitudes and behavior of the avatar they are represented by (i.e., the Proteus effect; e.g., Yee & Bailenson, 2007; Yee, Bailenson, & Ducheneaut, 2009; Peña, Hancock, & Merola, 2009). We consider this a demonstration of feature migration in the sense of Ma et al. (2016): increasing the overlap of self- and other-representation invites illusionary conjunctions, in which features of the other become features of oneself. Moreover, enfacing an ape tempted participants to attribute more emotions to apes, another case of feature migration: when becoming more like an ape, participants took their emotional capabilities with them, so to speak. Note that synchrony affected primary and secondary emotions alike. While primary emotions are considered to be shared among humans and other highly evolved animals, secondary emotions are commonly thought to be uniquely human (Epstein, 1984). If our human participants would have judged apes from their human perspective, one might have expected that synchrony has a stronger impact on primary emotions attributed to apes than on secondary emotions. However, given that synchrony enhanced the attribution of both kinds of emotions, it seems that the emotion attribution in this condition relied on a more “insider perspective”, that is, from the perspective of an ape feeling like a human. In other words, when embodying another person (or a member of another species), self-related attributes can be extended to the embodied others who become more like the self. This fits with the observation that synchrony had no effect on the attribution of emotions to humans, which also rules out the possibility that the enfacement experience facilitated emotion attribution in general.

In the present study, we were interested to see whether feature migration can lead to the integration of features of another individual into the representation of oneself. However, we would like to emphasize that, theoretically speaking, features would be expected to migrate both ways: from other to self and from self to other. So, while our present study was tapping into the route from other to self, some recent studies have provided evidence that features may migrate from self to other as well. For instance, body-ownership illusions have been found to induce a more positive attitude toward social out-groups (e.g., Maister et al., 2015), which from our theoretical view might suggest that a positive self-image can migrate to an embodied other. Indeed, considering that attributing emotions to outgroup members represents an attitude, our findings are consistent with, and can be seen as an extension of previous outgroup studies along the lines of Maister et al. (2015). As already mentioned, synchronous stimulation of participants’ own hand and a rubber hand typical for a racial outgroup member increased perceived ownership for the rubber hand and induced a more positive attitude toward that outgroup (Farmer et al., 2013). Similarly, Inzlicht, Gutsell, and Legault (2012) reported that negative attitudes toward a racial outgroup were reduced by synchronizing one’s own movements with those of an outgroup member. It is true that such a modulation of implicit racial attitudes has not been observed in a recent study using a static enfacement paradigm (Estudillo & Bindemann, 2016). This suggests that embodiment illusions are not always effective in biasing self- and/or other-perception, especially when concerning body parts that are strongly tied to the self-identity, just like faces. However, recent comparisons between dynamic and static hand illusion conditions have revealed that dynamic conditions, as used in the present study, are much more sensitive to manipulations and strongly increase the coherence between dependent measures (Ma & Hommel, 2015a, b). It is thus possible that dynamic conditions, perhaps together with the morphing technique we used in the present study, will be more successful in demonstrating modifications of racial attitudes.

Note that in the present study we relied on explicit measures but did not use implicit measures of ownership, a decision we made for two reasons. First, explicit and implicit measures of body ownership have often been demonstrated to dissociate (e.g., Liepelt, Dolk & Hommel, 2017; Ma & Hommel, 2015a, b). This implies that explicit and implicit measures rely on different kinds of information, and so far, no theoretical account for such dissociations has been suggested. This renders it unclear what kind of information implicit measures would add and which theoretical implications the convergence or divergence with explicit measures would have. Second, the inclusion of implicit measures was unlikely to be successful in our study. Previous face-ownership studies have used self–other discrimination or recognition of self–other morphed faces (e.g., Sforza et al., 2010; Tsakiris, 2008; Tajadura-Jiménez et al., 2012) as an implicit measure. The typical outcome was the tendency of participants to under-discriminate between pictures of oneself and of a very similar human other in the synchrony, compared to the asynchrony condition. This bias is commonly very small (around 5%), which renders it extremely unlikely to get anything measurable when comparing oneself against the picture of an ape.

Another important consideration pertains to the fact we did not assess participants’ stereotypes towards apes to verify whether and to what degree the common stereotype that humans are more intelligent than nonhuman animals was shared by our participants. As we pointed out, explicitly assessing this stereotype would have been likely to introduce unwelcome confounds. And yet, it would be interesting to test whether individual differences in the degree to which participants consider apes as less intelligent would predict the sizes of these synchrony-induced changes in intelligence and emotional competence judgments. Future studies might either assess these stereotypes in separate sessions and at considerable temporal distance and/or assess them in a more indirect fashion. Moreover, it would be interesting to see whether interventions of the sort investigated in the present study would lead to longer lasting changes in individual stereotypes—as suggested by the observations of Farmer et al. (2013) and Inzlicht et al. (2012). Notwithstanding these interesting open questions, our findings provide further evidence that the boundaries between perceived self and perceived other are rather flexible, and that representational self–other overlap invites illusory conjunctions of features from one representation to the other—including others of another species.

## References

[CR1] Ahn SJ, Bostick J, Ogle E, Nowak K, McGillicuddy K, Bailenson JN (2016). Experiencing nature: Embodying animals in immersive virtual environments increases inclusion of nature in self and involvement with nature. Journal of Computer-Mediated Communication.

[CR2] Aron A, Aron EN, Smollan D (1992). Inclusion of other in the self scale and the structure of interpersonal closeness. Journal of Personality and Social Psychology.

[CR3] Banakou D, Groten R, Slater M (2013). Illusory ownership of a virtual child body causes overestimation of object sizes and implicit attitude changes. Proceedings of the National Academy of Sciences.

[CR4] Baumeister RE, Bratslavsky E, Muraven M, Tice DM (1998). Ego depletion: Is the active self a limited resource?. Journal of Personality and Social Psychology.

[CR5] Botvinick M, Cohen J (1998). Rubber hands ‘feel’ touch that eyes see. Nature.

[CR6] Bufalari I, Lenggenhager B, Porciello G, Serra HB, Aglioti SM (2014). Enfacing others but only if they are nice to you. Frontiers in Behavioral Neuroscience.

[CR7] Chartrand TL, Bargh JA (1999). The chameleon effect: The perception-behavior link and social interaction. Journal of Personality and Social Psychology.

[CR8] Colzato LS, de Bruijn ER, Hommel B (2012). Up to “me” or up to “us”? The impact of self-construal priming on cognitive self-other integration. Frontiers in Psychology.

[CR9] De Dreu CK, Greer LL, Van Kleef GA, Shalvi S, Handgraaf MJ (2011). Oxytocin promotes human ethnocentrism. Proceedings of the National Academy of Sciences.

[CR10] Demoulin S, Leyens JP, Paladino MP, Rodriguez-Torres R, Rodriguez-Perez A, Dovidio J (2004). Dimensions of “uniquely” and “non-uniquely” human emotions. Cognition and Emotion.

[CR11] Epstein S (1984). Controversial issues in emotion theory. Review of Personality and Social Psychology.

[CR12] Estudillo AJ, Bindemann M (2016). Multisensory stimulation with other-race faces and the reduction of racial prejudice. Consciousness and Cognition.

[CR13] Farmer H, Maister L, Tsakiris M (2013). Change my body, change my mind: the effects of illusory ownership of an outgroup hand on implicit attitudes toward that outgroup. Frontiers in Psychology.

[CR14] Gallagher S (2000). Philosophical conceptions of the self: Implications for cognitive science. Trends in Cognitive Sciences.

[CR15] Glass GV, Peckham PD, Sanders JR (1972). Consequences of failure to meet assumptions underlying the fixed effects analyses of variance and covariance. Review of Educational Research.

[CR16] Govorun O, Payne BK (2006). Ego—depletion and prejudice: Separating automatic and controlled components. Social Cognition.

[CR17] Graziano MSA, Botvinick MM, Prinz W, Hommel B (2002). How the brain represents the body: insights from neurophysiology and psychology. Common mechanisms in perception and action: Attention and Performance XIX.

[CR18] Harwell MR, Rubinstein EN, Hayes WS, Olds CC (1992). Summarizing Monte Carlo results in methodological research: The one-and two-factor fixed effects ANOVA cases. Journal of Educational Statistics.

[CR19] Hershfield HE, Goldstein DG, Sharpe WF, Fox J, Yeykelis L, Carstensen LL, Bailenson JN (2011). Increasing saving behavior through age-progressed renderings of the future self. Journal of Marketing Research.

[CR20] Hommel B (2004). Event files: Feature binding in and across perception and action. Trends in Cognitive Sciences.

[CR21] Hommel B, Colzato LS, van den Wildenberg WPM (2009). How social are task representations?. Psychological Science.

[CR22] Hommel B, Müsseler J, Aschersleben G, Prinz W (2001). The theory of event coding (TEC): A framework for perception and action planning. Behavioral & Brain Sciences.

[CR23] Inzlicht M, Gutsell JN, Legault L (2012). Mimicry reduces racial prejudice. Journal of Experimental Social Psychology.

[CR24] Jones BC, DeBruine LM, Little AC, Conway CA, Feinberg DR (2006). Integrating gaze direction and expression in preferences for attractive faces. Psychological Science.

[CR25] Kalckert A, Ehrsson HH (2014). The moving rubber hand illusion revisited: Comparing movements and visuotactile stimulation to induce illusory ownership. Consciousness and Cognition.

[CR26] Kilteni K, Grau-Sánchez J, De Las Heras MV, Rodríguez-Fornells A, Slater M (2016). Decreased corticospinal excitability after the illusion of missing part of the arm. Frontiers in Human Neuroscience.

[CR27] Kilteni K, Normand JM, Sanchez-Vives MV, Slater M (2012). Extending body space in immersive virtual reality: a very long arm illusion. PLoS One.

[CR28] Kühnen U, Oyserman D (2002). Thinking about the self influences thinking in general: Cognitive consequences of salient self-concept. Journal of Experimental Social Psychology.

[CR29] Lenggenhager B, Tadi T, Metzinger T, Blanke O (2007). Video ergo sum: manipulating bodily self-consciousness. Science.

[CR30] Liepelt R, Dolk T, Hommel B (2017). Self-perception beyond the body: The role of past agency. Psychological Research Psychologische Forschung.

[CR31] Ma K, Hommel B (2015). Body-ownership for actively operated non-corporeal objects. Consciousness and Cognition.

[CR32] Ma K, Hommel B (2015). The role of agency for perceived ownership in the virtual hand illusion. Consciousness and Cognition.

[CR33] Ma K, Sellaro R, Lippelt DP, Hommel B (2016). Mood migration: How enfacing a smile makes you happier. Cognition.

[CR34] Maister L, Sebanz N, Knoblich G, Tsakiris M (2013). Experiencing ownership over a dark-skinned body reduces implicit racial bias. Cognition.

[CR35] Maister L, Slater M, Sanchez-Vives MV, Tsakiris M (2015). Changing bodies changes minds: owning another body affects social cognition. Trends in Cognitive Sciences.

[CR36] Memelink J, Hommel B (2013). Intentional weighting: A basic principle in cognitive control. Psychological Research Psychologische Forschung.

[CR37] Oh SY, Bailenson J, Weisz E, Zaki J (2016). Virtually old: Embodied perspective taking and the reduction of ageism under threat. Computers in Human Behavior.

[CR38] Peck TC, Seinfeld S, Aglioti SM, Slater M (2013). Putting yourself in the skin of a black avatar reduces implicit racial bias. Consciousness and Cognition.

[CR39] Peña J, Hancock JT, Merola NA (2009). The priming effects of avatars in virtual settings. Communication Research.

[CR40] Piryankova IV, Wong HY, Linkenauger SA, Stinson C, Longo MR, Bülthoff HH, Mohler BJ (2014). Owning an overweight or underweight body: distinguishing the physical, experienced and virtual body. PLoS One.

[CR41] Porciello, G., Bufalari, I., Minio-Paluello, I., Di Pace, E., & Aglioti, S. M. The ‘Enfacement’illusion: A window on the plasticity of the self. *Cortex*. (**in press**) 10.1016/j.cortex.2018.01.007 29478669

[CR42] Raven JC (1938). Progressive matrices: A perceptual test of intelligence.

[CR43] Rosenberg RS, Baughman SL, Bailenson JN (2013). Virtual superheroes: Using superpowers in virtual reality to encourage prosocial behavior. PLoS One.

[CR44] Schmider E, Ziegler M, Danay E, Beyer L, Bühner M (2010). Is it really robust? Reinvestigating the robustness of ANOVA against violations of the normal distribution assumption. Methodology: European Journal of Research Methods for the Behavioral and Social Sciences.

[CR45] Sforza A, Bufalari I, Haggard P, Aglioti SM (2010). My face in yours: Visuo-tactile facial stimulation influences sense of identity. Social Neuroscience.

[CR46] Shimada S, Fukuda K, Hiraki K (2009). Rubber hand illusion under delayed visual feedback. PLoS One.

[CR47] Simmons JP, Nelson LD, Simonsohn U (2011). False-positive psychology: Undisclosed flexibility in data collection and analysis allow presenting anything as significant. Psychological Science.

[CR48] Slater M, Perez-Marcos D, Ehrsson HH, Sanchez-Vives MV (2008). Towards a digital body: the virtual arm illusion. Frontiers in Human Neuroscience.

[CR49] Slater M, Spanlang B, Sanchez-Vives MV, Blanke O (2010). First person experience of body transfer in virtual reality. PLoS One.

[CR50] Steptoe W, Steed A, Slater M (2013). Human tails: ownership and control of extended humanoid avatars. IEEE Transactions on Visualization and Computer Graphics.

[CR51] Suma EA, Krum DM, Lange B, Koenig S, Rizzo A, Bolas M (2013). Adapting user interfaces for gestural interaction with the flexible action and articulated skeleton toolkit. Computers & Graphics.

[CR52] Tajadura-Jiménez A, Grehl S, Tsakiris M (2012). The other in me: interpersonal multisensory stimulation changes the mental representation of the self. PLoS One.

[CR53] Tajfel H, Turner JC (1986). The social identity theory of intergroup behavior. Psychology of Intergroup Relations.

[CR54] Treisman AM, Gelade G (1980). A feature-integration theory of attention. Cognitive Psychology.

[CR55] Tsakiris M (2008). Looking for myself: Current multisensory input alters self-face recognition. PLoS One.

[CR56] Tsakiris M, Carpenter L, James D, Fotopoulou A (2010). Hands only illusion: Multisensory integration elicits sense of ownership for body parts but not for non-corporeal objects. Experimental Brain Research.

[CR57] van der Hoort B, Guterstam A, Ehrsson HH (2011). Being Barbie: the size of one’s own body determines the perceived size of the world. PLoS One.

[CR58] Vohs KD, Ciarocco N, Baumeister R, Vohs K (2004). Interpersonal functioning requires self-regulation. Handbook of self-regulation: Research, theory, and applications.

[CR60] Yee N, Bailenson JN (2006). Walk a mile in digital shoes: The impact of embodied perspective-taking on the reduction of negative stereotyping in immersive virtual environments. Proceedings of PRESENCE 2006: The Ninth Annual International Workshop on Presence (Cleveland, OH).

[CR59] Yee N, Bailenson J (2007). The Proteus effect: The effect of transformed self-representation on behavior. Human Communication Research.

[CR61] Yee N, Bailenson JN, Ducheneaut N (2009). The Proteus effect: Implications of transformed digital self-representation on online and offline behavior. Communication Research.

